# Reproductive health and lifestyle factors associated with health-related quality of life among perinatally HIV-infected adolescents in Uganda

**DOI:** 10.1186/s12955-015-0366-6

**Published:** 2015-10-21

**Authors:** Scovia Nalugo Mbalinda, Noah Kiwanuka, Dan K. Kaye, Lars E. Eriksson

**Affiliations:** Department of Nursing, School of Health Sciences, Makerere University, P.O.Box 7072, Kampala, Uganda; Department of Epidemiology and Biostatistics, School of Public Health, College of Health Sciences, Makerere University, Kampala, Uganda; Department of Obstetrics and Gynecology, College of Health Sciences, Makerere University, Kampala, Uganda; Department of Learning, Informatics, Management and Ethics, Karolinska Institutet, Solna, Sweden; Department of Infectious Diseases, Karolinska University Hospital, Stockholm, Sweden; School of Health Sciences, City University London, London, UK

**Keywords:** HIV, Health related quality of life, Sexual and reproductive health, Adolescents

## Abstract

**Background:**

With increased survival of perinatally HIV - infected adolescents due to antiretroviral therapy (ART), the focus of HIV care has shifted to health-related quality of life (HRQoL) as a measure of disease progression, effects of ART co-morbidity and prognosis. We assessed factors associated with better HRQoL in perinatally HIV -infected adolescents in Uganda by determining the associations between sexual and reproductive health (SRH) or lifestyle experiences on HRQoL.

**Methods:**

In a cross-sectional study, data on SRH, lifestyle experiences, socio demographic factors, communication with parents on sexuality and satisfaction of SRH services in ART clinics were collected from 614 HIV perinatally infected adolescents aged 10–19 using an interviewer-administered survey questionnaire. HRQoL data were collected using the Medical Outcomes Study HIV Health Survey instrument (MOS-HIV). Factors associated with better HRQoL were analysed using multiple logistic regression.

**Results:**

The mean age was 16.2 ± 2.1 years, 362 (58.8 %) were females and 210 (34.2 %) were sexually active. Adolescents on ART were twice likely to present with better physical health (AOR = 2.07, 95 % CI: 1.24–3.46) and four times more likely to present with better mental health (AOR = 3.9, 95 % CI: 2.22–6.92) than those who were not on ART. There were no statistically significant associations between SRH (ever had sex, ever been pregnant, condom use, contraceptive use) or life style factors and physical health or mental health. Those with secondary or tertiary education were more likely to present with a better mental health (AOR = 5.3, 95 % CI: 1.86–15.41) compared those who had attained primary or no education. Participants who desired to have a child in future more likely (AOR 1.7, 95 % CI: 1.05–3.00) to present with a better mental health. Lack of communication with guardians on sexuality (AOR = 0.6, 95 % CI: 0.40–0.89), or dissatisfaction with SRH services (AOR 0.34, 95 % CI: 0.18–0.62) were associated with poorer mental health.

**Conclusion:**

Among perinatally HIV-infected adolescents in Uganda, being on ART was associated with better physical and mental health while lack of communication with guardians on sexuality or dissatisfaction with SRH services was associated with poor mental health. Adolescents with pregnancy intentions were more likely to have a better mental health.

## Background

Globally, it is estimated that about 2.1 million adolescents aged 10–19 years are HIV positive [1]. The majority of these adolescents acquired HIV perinatally from their HIV infected mothers during pregnancy or birth or when they were breastfed. Nearly 2 million of these adolescents live in sub-Saharan Africa, 70 % of whom live in South Africa, Nigeria, Kenya, Tanzania, Uganda, Zimbabwe, Mozambique, Zambia, Ethiopia and Malawi [1]. In the year 2012, about 260,000 new HIV infections occurred in children worldwide [2]. With improved access to antiretroviral therapy (ART), most of these children will soon reach adolescence. In Uganda, the overall HIV prevalence among adolescents aged 15–19 years increased from 1.5 % in 2004–2005 to 2.4 % in 2011, while the overall national prevalence increased from 6.4 to 7.3 % [3]. In 2012, an estimated 110,000 adolescents were living with HIV in the country [4].

In general, quality of life (QOL) is the perceived quality of an individual’s daily life assessment of their well-being or lack thereof. QOL includes all emotional, social, and physical aspects of the individual’s life. In health care, health-related quality of life (HRQoL) is an assessment of how the individual’s well-being may be affected over time by a disease, a disability, or a disorder [5]. People living with HIV have significantly lower HRQoL than the general population, despite being virologically and immunologically stable [6].

The challenges that perinatally HIV-infected adolescents must deal with include; living in constant fear of having their serostatus being found out by peers [7–10], resentment and anger towards their parents, uncertainty about living a normal life and having a family in the future, treatment fatigue, as well as potential presence of feelings of powerlessness, hopelessness, depression and in extreme cases, suicidal ideation [11, 12]. These may affect their HRQoL. Many perinatally HIV-infected adolescents face the problem of orphan hood by having lost their parents or guardians, thereafter taking responsibility for welfare of younger siblings or other ill family members, stigma and discrimination, the fear of being viewed as abnormal, and being confronted with fear of death and an uncertain future [13, 14]. These may affect their HRQoL. Other challenges include living with an incurable chronic disease, lifelong medication dependence and adherence issues, drug-related toxicity and unwanted effects [11, 15–19]. All these may also affect the HRQoL. Consequently, some adolescents will have mental problems due to their HIV status or the treatment for HIV [16, 20, 21]. This, in turn, have potential implications related to their willingness to seek medical care, and motivation to adhere to therapy and ultimately influence health outcomes [22].

Several factors have been shown associated with HRQoL in patients with HIV, these include; Physical manifestations, antiretroviral therapy, psychological well-being, social support systems, coping strategies, spiritual well-being, and psychiatric comorbidities [23, 24]. Perinatally HIV-infected adolescents, like other adolescents form relationships, and become sexually active [25, 26]. Some relationships could be positive and supportive which may improve HRQoL. Yet, other relationships may be negative which negatively affect HRQoL. With increased survival due to ART, there is increasing survival of children (with a slowly progressive HIV infection) who are now reaching physiological and psychological maturation. As a consequence, they are at risk of becoming pregnant or causing a pregnancy, acquiring sexually transmitted infections and transmitting HIV [27]. Pregnancy and acquisition of an STI may also affect their HRQoL. Pregnancy related and STI comorbidities and associated physical symptoms may affect HRQoL. Disclosure of unwanted pregnancy may lead to denial from the partner which may affect the mental health of these adolescents [28, 29]. On the other hand, a pregnancy may be positive and acceptable by partner or their family, which improves the quality of life of the adolescents [30]. Additionally, adolescents living in constant fear of death may view pregnancy as a way of living a successor [31]. In addition to physical morbidity, STIs may lead to suspicion and mistrust in the relationships [32], thus leading to potential effect on quality of life. Lastly, ART treatment may improve immunity and reduce risk of co-morbidity from opportunistic infections, thus improving quality of life [17]. However, side-effects of ART may worsen quality of life [33].

With effective treatment available, the focus of HIV care has shifted to HRQoL, as a measure of disease progression, effects of ART and co-morbidity and prognosis [34, 35]. Focus should therefore be on assessment of HRQoL among HIV infected adults as well as adolescents in daily care [18, 34, 35]. A routine assessment of HRQoL in persons with HIV infection has the potential to improve care by assessing and monitoring treatment effects, enhancing communication between patient and provider, and tracking changes in functional status over time. There is limited information on association between sexual and reproductive health (SRH) or life style factors and HRQoL among Ugandan adolescents. Therefore, the aims of the present study were to describe HRQoL and to assess the association between SRH or lifestyle experiences and HRQoL among perinatally HIV infected adolescents in Uganda.

## Methods

### Study setting and sampling of clinics

This cross sectional study was conducted in the eastern, western and northern regions of Uganda. Twelve health facilities that provide ART were selected (four public and nonprofit private health facilities from each region). A multi-stage random sampling method was employed. Using the Ministry of Health (MOH) documents (2011), all health units with ≥50 clients aged 0–15 years were included in the sampling frame. This is because the MOH data is captured using 0–15 years and 15 years and above. The regions were purposively selected because the majority of the area is rural, whereas the previous studies [25, 36] on perinatally HIV infected adolescents were performed in the central region. Three facilities were selected by simple random sampling and one regional hospital was purposively selected since it is the referral hospital of the region. Each of the clinics provides ART and counseling regarding drug adherence, and general care.

### Participants

Perinatally HIV-infected adolescents aged 10–19 years were recruited between September 2013 and March 2014. The sample size was calculated using Kish Leslie formula [37]. We assumed probability of poor HRQoL of 50 %, an acceptable error of 5 %, and Z value of 1.96 and a design effect of two. To obtain a final estimated sample size of 672, the goal was to recruit 56 participants consecutively from each health facility.

### Data collection methods

**Fig. 1:**
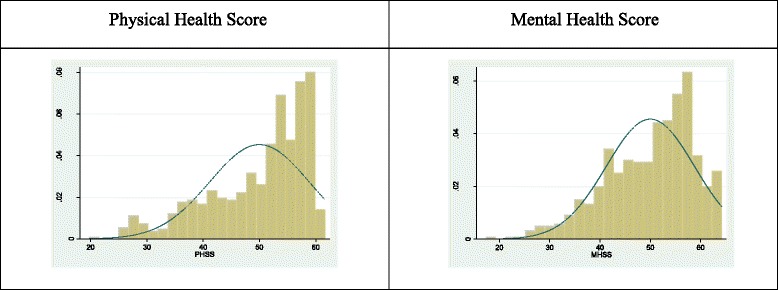
Distribution of physical and mental scores after Principal Component Analysis

Data were collected using interviewer-administered questionnaires that included questions about socio-demographic characteristics, sexuality, disclosure of HIV sero-status, sexually transmitted infections, contraception, pregnancy and procreation intentions, parental communication and sex education, peer influence and access and utilization of SRH services. To measure HRQoL, the Medical Outcomes Study HIV Health Survey instrument (MOS-HIV) was used [38]. We used an English version which has been culturally adapted, translated and validated in Uganda [39]. This English version was translated to four major languages (Lusoga, Ateso, Luo and Runyakitara) where the adolescents were recruited. The participants who were able to speak English responded in English (Majority) and those who did not know English responded in their respective languages. These languages are spoken widely in the areas where the data was collected. The tool includes 35 questions that assess eleven dimensions of health including; General Health Perceptions (GHP), Pain (P), Physical Functioning (PF), Role Functioning (RF), Social Functioning (SF), Mental Health (MH), Energy/Fatigue (EF), Health Distress (HD), Cognitive Function (CF), Quality of Life (QL) and Health Transition (HT). All items are answered on a scale of two, three, five or six response options. The MOS-HIV subscales are scored as summated rating scales and transformed on a 0–100 scale where higher scores indicate better health. Furthermore, a Physical Health Summary score (PHSS) and a Mental Health Summary score (MHSS), both on a 0–100 scale, can be generated by standardizing the scores using the weighting coefficients estimated in a previous study and provided by the author of the questionnaire [40, 41]. The data was not normally distributed, so we used the median instead of mean as a cut off for better and poorer HRQoL in the PHS and MHS (Fig. 1). Both scores were dichotomized at their respective medians yielding ordinal binary better and poorer HRQoL.

### Data analysis

Demographics characteristics were sumararised in frequency and percentage. Participant’s age was grouped into 10–14 years and 15–19 years. Education status was grouped into in school and out of school and education level was grouped into three categories: no education, primary and secondary. Occupation was grouped into three categories: students, unemployed and volunteers (employed). Living with parents was grouped into; one parent-means staying with at least one parent and not staying with a parent means not staying with any parent.

The outcome variable was better HRQoL as measured by the MOS-HIV instrument. The independent variables were socio-demographic characteristics (age, sex, education status, level of education hoped to complete and distance to clinic), medical factors (ART, disclosure of HIV status and comorbidities) SRH (ever had sex, pregnancy, condom use, contraceptive use, utilization of SRH services and parent communication on sexuality) and life style factors (smoking and alcohol use). To determine factors associated with better HRQoL on the PHSS and MHSS, we used the median as a cut-off as described previously. The outcome was defined as 1 if scores were greater or equal to the median and 0 if scores were below the median. Bivariate logistic regression analysis was carried out to determine associations between the outcome variable (HRQoL) and the independent factors. After checking for collinearity, all variables with a *P* value of ≤0.2 at the bivariate analysis were included in the multiple logistic regression models. During model development, all predictor variables with a *p*-value of ≤0.2 [42] at bivariate analysis were considered for inclusion in the multivariate logistic regression model. We used the likelihood ratio test to check for model fit by dropping insignificant variables first and refit the model until we got the best fit. Collinearity was assessed using a correlation matrix and cross-checked by the use of variance inflation factor which was set at ten [43]. In case two variables were associated (*P* <0.05), the variable explaining the largest variability (smaller p value at univariate analysis) was retained. Significance was set at 0.05 and all of the analyses were two tailed. Analyses were done using STATA®12.

### Ethical consideration

Ethical approval was obtained from the Higher Degrees and Research Ethics committee, College of Health Sciences, Makerere University and Uganda National Council for Science and Technology (REC REF 2012-085). Administrative clearance and permissions were obtained from the management of the health facilities. Both written and verbal consents were obtained from adolescents above 18 years. For adolescents below 18 years, assent as well as consent from parents or guardians was obtained. Participation was voluntary and all the interviews were conducted in privacy and participant’s confidentiality was maintained throughout the study by giving an identification number on the questionnaire.

## Results

**Fig. 2:**
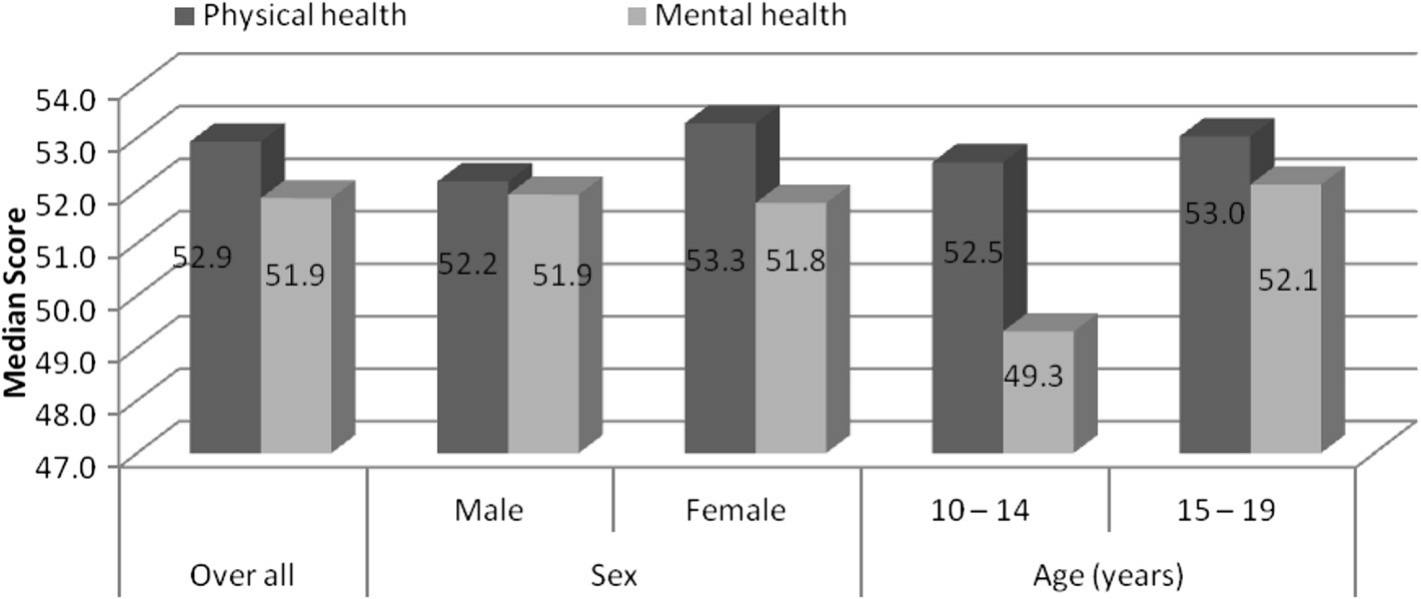
Median scores for PHS and MHS of the sample of HIV perinatally infected adolescents (*n* = 614)

Table 1 summarizes the characteristics of 614 participants. Of these 361(58.8 %) were males. The mean, standard deviation (SD) and median, interquartile range (IQR) age for the participants were 16.2 (2.06) and 16 (15–18) years respectively. For males, the mean age was 15.9 (SD 2.3) and for females 16.4 (SD 1.8) years. Overall, 500 (81.4 %) were aged 15–19 years, 455 (74.1 %) were enrolled in school and 374 (60.9 %) hoped to complete tertiary education; and 269 (43.8 %) had lost both parents. The median PHSS was 52.9 (IQR 44.6–57.4) and MHSS was 51.5 (IQR 44.0–56.9) for the whole sample. Figure 2 shows median PHSS and MHSS for the whole sample, males and females and different age groups. The difference between males and females and age groups with respect to summary scores was not statistically significant.

**Table 1:** Descriptive statistics on the sample of HIV perinatally infected adolescents (*n* = 614)

Variables		Frequency	Percentage
Sex	Male	253	41.2
	Female	361	58.8
Age groups	10 – 14 years	114	18.6
	15 – 19 years	500	81.4
Religion	Catholic	257	41.9
	Protestant	247	40.2
	Others	110	17.9
Education status	Out of school	159	25.9
	In school	455	74.1
Highest Level of education attained	None	29	4.7
	Primary level	355	57.2
	Secondary level	230	37.5
Highest level of education hoped to complete	Primary	63	10.3
	Secondary	106	17.3
	Tertiary	374	60.9
	Don’t know	71	11.6
Occupation	Students	455	74.1
	Volunteers	49	8
	Stays home	110	17.9
Region	Western	229	37.3
	Eastern	172	28
	Northern	213	34.7
Distance to clinic	Within 5 km	193	31.4
	More than 5 km	421	68.6
Living with parents	Both parents	103	16.8
	One parent	243	39.4
	Not staying with any parents	268	43.6

Table 2 shows unadjusted association between socio demographics characteristics and PHSS and MHSS. Adolescents in school were more likely (OR: 1.53, 95 % CI 1.06–2.20) to present with better mental health than those out of school. Adolescents in secondary level of education were 9 times (OR: 9.44, 95 % CI 3. 28.04) and 5 times (OR: 5.48, 95 % CI 2.15–13.99) more likely to present with a better mental health and physical health respectively compared to those adolescents with no formal education. Adolescents who were unemployed were less likely to present with better mental health (OR: 0.46, 95 % CI 0.29–0.74) than those adolescents who were employed.

**Table 2:** Unadjusted odds ratios of socio-demographics factors associated with PHS and MHS

		Physical health			Mental health		
		Crude OR	95 % CI	*P*	Crude OR	95 % CI	*P*
Sex	Male	Ref					
	Female	1.22	0.89 –1.69	0.219	0.99	0.72 – 1.36	0.94
Age groups	10 – 14 years	Ref					
	15 – 19 years	1.04	0.70 – 1.57	0.836	1.35	0.90 – 2.04	0.15
Religion	Catholic	Ref					
	Protestant	1.30	0.91 – 1.84	0.15	1.38	0.97 – 1.96	0.07
	Others	1.39	0.87 – 2.19	0.15	1.44	1.91 – 2.26	0.12
Education status	Out of school	Ref					
	In school	1.38	0.96 – 1.99	0.081	1.53	1.06 – 2.20	0.02
Level of education	None	Ref					
	Primary level	3.38	1.34 – 8.51	0.01	5.27	1.79 – 15.45	0.00
	Secondary level	5.48	2.15 – 13.99	0.00	9.44	3.18 – 28.04	0.00
Occupation	Students	Ref					
	Volunteers	0.82	0.45 – 1.49	0.509	1.21	0.66 – 2.22	0.54
	Stays home	0.82	0.52 – 1.31	0.416	0.46	0.29 – 0.75	0.00
Region	Western	Ref					
	Eastern	0.26	0.17 – 0.39	0.00	0.69	0.47 – 1.03	0.07
	Northern	1.1	0.76 – 1.62	0.595	0.61	0.42 – 0.89	0.01
Distance to clinic	Within 5 km	Ref					
	More than 5 km	0.68	0.49 – 0.96	0.03	0.90	0.64 – 1.26	0.52
α Living with parents	Both parents	Ref					
	One parent	1.09	0.69 – 1.74	0.69	0.96	0.61 – 1.53	0.88
	Not staying with a parent	1.46	0.87 – 2.16	0.17	0.90	0.63 – 1.56	0.96

α Living with parents (whether the adolescent is living with parent) - One parent-means staying with at least one parent and Not staying with a parent means not staying with any parent

Table 3 shows unadjusted association between medical, SRH characteristics with PHSS and MHSS. Adolescents on ARV were twice more likely to present with better physical health (OR: 2.3, 95 % CI 1.47–4.11) and four times more likely to present with better mental health (OR: 4.11, 95 % CI 2.50–6.77) as compared to those not on ARV. The adolescents who had one sexual partner were more likely to present with better mental health (OR: 1.94, 95 % CI 1.00–3.74) compared to those with two or more sexual partners. Adolescents who were not communicating with their guardians on sexuality issues were less likely (OR: 0.69, 95 % CI 0.50–0.97) to present with better mental health compared to those who talked with their guardians about sexuality issues. The adolescents who were not smoking were nearly twice likely (OR 1.7, 95 % CI: 1.09–2.74) present with better physical health than those adolescents who were smoking.

**Table 3:** Unadjusted odds ratios of SRH and life style factors associated with PHS and MHS

		PHS			MHS		
		Crude OR	95 % CI	*P*	Crude OR	95 % CI	*P*
Currently on ARVs	No	Ref	1.47–3.62	0.00	4.11	2.50–6.77	0.00
	Yes	2.3					
Ever had sex	No	Ref	0.62–1.20	0.395	0.92	0.65–1.28	0.61
	Yes	0.87					
Age at first sex	<16 years	0.95	0.54–1.64	0.851	0.82	0.47–1.42	0.48
	≥16 years						
Number of sexual partners	None	Ref				1.00–3.74	0.05
	One	1.26	0.66–2.40	0.48	1.9	0.71–4.06	0.20
	>1 more	1.52	0.64–3.59	0.34	1.7		
Ever been married	No	Ref					
	Yes	1.79	0.93–3.45	0.084	0.6	0.31–1.18	0.14
Ever disclosed HIV status	No	Ref					
	Yes	1.06	0.67–1.68	0.802	1.25	0.79–1.9	0.34
Ever been treated for an STD/STI	No	1	0.52–1.23	0.324	1.14	0.74–1.75	0.56
	Yes	0.8					
Using family planning	No	Ref	0.57–1.18	0.287	1.19	0.83–1.71	0.33
	Yes	1.07					
Ever been pregnant	No	Ref	0.65–1.74	0.8	0.78	0.48–1.28	0.33
	Yes	1.07					
Wants to have child in the future	No	Ref	0.52–1.24	0.327	1.51	0.97–2.34	0.06
	Yes	0.8					
Parent adolescent communication on sexuality.	Yes	Ref	0.55–1.06	0.106	0.69	0.50–0.97	0.03
	No	0.76					
Ever smoked cigarette	No	Ref	1.09–2.74	0.02	2.05	0.76–5.52	0.15
	Yes	1.7					
Ever drunk alcohol	No	Ref	0.41–1.07	0.10	1.75	0.76–2.02	0.34
	Yes	0.67					
Having a friend who is smoker	No	Ref	0.96–1.94	0.08	0.71	0.45–1.11	0.14
	Yes	1.3					
Having friend who is drinking alcohol	No	Ref					
	Yes	0.73	0.52–1.03	0.081	1.02	0.69–1.38	0.88
Satisfied with the SRH service	Yes	Ref	0.28–0.73	0.00	0.26	0.15–0.44	
	No	0.46					0.00

Table 4 shows the adjusted association of the independent variables and PHSS. Adolescents on ART were twice as likely to present with better physical health (AOR 2.07, 95 % CI: 1.24–3.46) compared to those who were not on ARVs. Furthermore, adolescents who had friends who smoked were less likely (AOR 0.48, 95 % CI: 0.29–0.80) to present with better physical health than those who did not have a friend who smoked. Adolescents recruited from the Northern region were less likely to present with better mental health (AOR 0.25, 95 % CI: 0.16–0.42) compared to adolescents from other regions.

**Table 4:** Adjusted odds ratios of SRH, life style factors and socio-demographic factors associated with PHS

Variables		PHSS		
	No (%)	AOR	95 % CI	*P*
Highest level of education hoped to complete				
Primary	63(10.3)	Ref		
Secondary	106((17.3)	0.41	0.20–0.85	0.01
Tertiary	374(60.9)	1.01	0.55–1.88	0.56
Don’t know	71(11.6)	0.67	0.31–1.50	0.34
Region				
Western	229(37.2)	Ref		
Eastern	172(28.0)	1.13	0.73–1.75	0.57
Northern	213(34.7)	0.25	0.16–0.42	<0.001
Distance to clinic				
Within 5 km	193(34.7)	Ref		
Within 20 km	421(68.6)	0.69	0.38–1.26	0.23
Currently on ARVs				
No	100	Ref		
Yes	513	2.07	1.24–3.36	Ref
Parent adolescent communication on sexuality				
No	224	Ref		
Yes	386	0.87	0.59–1.27	0.47
Has a friend who is smoking cigarette				
No	91	Ref		
Yes	520	0.48	0.29–0.80	<0.001
Satisfied with the SRH service				
No	88	Ref		
Yes	521	0.69	0.41–1.16	0.17

*AOR* adjusted odds ratio, adjusted for variables included in the model

Table 5 shows the adjusted association of the independent variables and MHSS. Adolescents in secondary school were five times more likely to present with better mental health than those who did not have any form of education (AOR 5.3, 95 % CI: 1.86–15.41). Adolescents recruited from the Northern region were less likely (AOR 0.50, 95 % CI: 0.32–0.78) to present with better mental health than those recruited from other regions. Adolescents on ART were four time more likely to present with a better mental health (AOR 3.9, 95 % CI: 2.22–6.9). Participants who desired to have a child in future were two times more likely (AOR 1.7, 95 % CI: 1.05–3.00) to present with a better mental health than those who never intended to have children in the future. Adolescents who never discussed with their guardians on sexuality issues were less likely (AOR 0.6, 95 % CI: 0.40–0.89), to present with a better mental health compared to those who discussed with their guardians. Adolescents who had friends who smoked were less likely (AOR 0.57, 95 % CI: 0.35–0.98) to present with a better mental health. Adolescents who were not satisfied with SRH services were less likely (AOR 0.34, 95 % CI: 0.18–0.62) to present with a better mental health.

**Table 5:** Adjusted odds ratios of SRH, life style, and socio demographic factors associated MHS

		MHSS		
		AOR	95 % CI	
Religion				
Catholic	257(41.9)	Ref		
Protestant	246(40.2)	1.2	0.77–1.76	0.46
Others	110(17.6)	1.7	0.93–4.74	0.25
Level of education.				
None	29(4.7)	Ref		
Primary	355(57.2)	3.3	1.18–9.38	0.02
Secondary	230(37.5)	5.3	1.86–15.41	<0.00
Region				
Western	229(37.30	Ref		
Eastern	172(28.0 %)	0.78	0.4–1.24	0.29
Northern	213(34.7 %)	0.50	0.32–0.78	<0.001
Distance to clinic				
<5 km	193(34.7)	Ref		
>5 km	421(68.6)	0.63	0.38–1.05	0.08
Living with parents				
Both parents	103(16.8)	Ref		
One parent	243(39.4)	0.85	0.50–1.44	0.54
Not staying with a parent	268(43.6)	0.87	0.51–1.48	0.61
Currently on ARVs				
No	100(16.3)	Ref		
Yes	513(83.7)	3.9	2.22–6.92	<0.001
Wants to have a child in the future				
No	97(16..3)	Ref		
Yes	498(83.7)	1.7	1.05–3.00	0.03
Parent and adolescent communicating on sexuality issues.				
Yes	224(36.7)	Ref		
No	386(63.3)	0.6	0.40–0.89	0.01
Friend who is smoking				
No	91 (14.9)	Ref		
Yes	520(85.1)	0.57	0.34–0.98	0.04
Satisfied with the SRH service				
Yes	92(15.0)	Ref		
No	521(85.0)	0.34	0.18–0.62	<0.00

*AOR* adjusted odds ratio, adjusted for variables included in the model

## Discussion

There were no associations between SRH (ever had sex, ever been pregnant, condom use, contraceptive use) or lifestyle factors and physical health. However, adolescents on ARV were twice more likely to present with a better physical and four times more likely to present with better mental health as compared to those not on ARV and adolescents living in the Northern region or who had a friend who smoked were more likely to present with a worse physical or mental health compared to those living in the Western region or who did not have a friend who smoked. Adolescents who wanted to present with children in the future were more likely to have a better mental health. Not communicate with guardians about sexuality or who were dissatisfied with SRH services was associated with poorer mental health.

ART is effective in improving the self-rated HRQoL by improving the immune status of the patients [44, 45]. Our study shows perinatally -infected adolescents on ART had better physical and mental health compared to those who were not on treatment. This is in line with other studies that show that people on ART have better HRQoL [17, 46, 47]. There is thus a critical need to provide ART for adolescents who acquire HIV perinatally since treatment improves both the physical and mental domains of HRQOL.

Life style factors were not associated with HRQoL. Smoking increases the risks of developing serious illnesses like chronic obstructive pulmonary diseases or non-compliance to HIV treatment plan [48, 49]. Although there was no association between smoking and HRQoL in the current study, the adolescents who had a friend who was smoking were less likely to have better physical or mental health. Second hand smoke exposure in studies with adults been shown to be associated with reduced HRQoL [50, 51] and causes cardiovascular diseases and lung cancer [52, 53]. This may not be any different from adolescents.

SRH experiences may be associated with HRQoL, with effects that may be positive or negative. Because adolescents who want to have a family have something positive they look forward to, they are more likely to have a better mental health [54, 55], especially if they conceive. This study showed that adolescents who wanted to have children in the future were more likely to present with a better mental health. However, HIV positive pregnant women may experience increased health distress and worse health transition during the antenatal period compared to HIV negative pregnant women, mainly because of decreased social support and concern of the physical health of their children [56, 57]. Some HIV positive women become pregnant to avoid the stigma associated with childlessness and being identified as being HIV positive in their communities [58]. Yet others who get pregnant or are sexually active face criticism from the community because of potential transmission of HIV to their children or transmission to other community members [59]. These issues may influence HRQoL.

Parent communication on sexuality issues influences young people’s sexual behavior, which in turn affects HRQoL [60]. In this study, adolescents who communicated less with their parents were less likely to present with better mental health. Parent communication is highly influential in adolescents’ behavior as it could help them to develop positive individual values and make sexually healthy decisions, thus translating into an influence on HRQoL. Studies in Tanzania and elsewhere have found that parental communication improves outcomes of SRH and HIV prevention [61–63], which may translate into better HRQoL. There is a need for interventions such as parenting skills building that might enable parents to improve their relationships with their maturing children. This would equip parents with the appropriate skills for positive guidance and communication of their children and enhance safe sexual behaviors and SRH outcomes.

The study showed that participants recruited from northern region of the country reported poorer physical and mental health. This may be due to the northern region is being a post conflict area and studies have shown that people in general have higher rates of depression [64, 65]. This may affect the health related quality of life.

The study had several limitations. Due to the fact that sexual behaviour was self-reported, there was a potential of recall bias or under-reporting of sexual behaviour. Participants may have forgotten or may be embarrassed to reveal information on their sexual activity, especially for the females, and males may have exaggerated on issues on sexuality [66]. There could be a selection bias because of the way units were selected, as only health units with large numbers were selected. The results of the study may only be generalizable to the study population or other similar settings. We did not stratify the findings depending on clinic attended; this may affect the health related quality of life due to the differences in quality of services or care offered in the individual ART clinic. However, additionally, the strength of this study was the large sample size of perinatally HIV-infected adolescents. Furthermore, this study attempted to link SRH and life style factors with HRQoL. Secondly, the study used a standardised tool (MOS-HIV) that has been culturally adapted. Thirdly, data was collected in three regions of the country and from referral hospitals that are fairly representative of the general population in Uganda.

In our study gender was not associated with quality of life. However studies have shown that gender affects health transition, perceived HRQoL, health distress, social functioning and role functioning in the adult population [67–69]. Future research on gender and age differences (and their interaction) may provide potentially useful information for planning interventions to improve QoL and mental health among people infected with HIV/AIDS especially in perinatally HIV-infected adolescents. In addition, this was a cross sectional study which cannot evaluate causal relationships. There is need to study the effect of SRH and life style factors on HRQoL in a prospective or retrospective longitudinal study so that we can understand the cause-and-effect relationship between SRH, life style factors and HRQoL. Such studies could assess the effect of HRQoL on SRH and life style factors. Furthermore, this study did not assess the effect clustering due to belonging to a particular ART clinic yet quality of services offered may affect HRQoL. Future studies should assess the effect of clustering on HRQoL.

## Conclusion

There was no association between SRH (ever had sex, ever been pregnant, condom use, contraceptive use) or life style factors and physical health. Adolescents on ARV were twice more likely to present with a better physical and four times more likely to present with mental health as compared to those not on ARV. Adolescents who wanted to present with children in the future were more likely to present with a better mental health . Poor mental health outcomes were associated with not having communication with guardians about sexuality or with their dissatisfaction with SRH services. There is need to design and integrate age appropriate and culturally valid screening and management of mental health in SRH services for perinatally HIV-infected adolescents.
